# A Distributed Stream Processing Middleware Framework for Real-Time Analysis of Heterogeneous Data on Big Data Platform: Case of Environmental Monitoring

**DOI:** 10.3390/s20113166

**Published:** 2020-06-03

**Authors:** Adeyinka Akanbi, Muthoni Masinde

**Affiliations:** Centre for Sustainable Smart Cities, Central University of Technology, Free State 9300, South Africa; emasinde@cut.ac.za

**Keywords:** big data, stream processing, middleware, Internet of Things, Apache *Kafka*, drought

## Abstract

In recent years, the application and wide adoption of Internet of Things (IoT)-based technologies have increased the proliferation of monitoring systems, which has consequently exponentially increased the amounts of heterogeneous data generated. Processing and analysing the massive amount of data produced is cumbersome and gradually moving from classical ‘batch’ processing—extract, transform, load (ETL) technique to real-time processing. For instance, in environmental monitoring and management domain, time-series data and historical dataset are crucial for prediction models. However, the environmental monitoring domain still utilises legacy systems, which complicates the real-time analysis of the essential data, integration with big data platforms and reliance on batch processing. Herein, as a solution, a distributed stream processing middleware framework for real-time analysis of heterogeneous environmental monitoring and management data is presented and tested on a cluster using open source technologies in a big data environment. The system ingests datasets from legacy systems and sensor data from heterogeneous automated weather systems irrespective of the data types to Apache *Kafka* topics using *Kafka* Connect APIs for processing by the *Kafka* streaming processing engine. The stream processing engine executes the predictive numerical models and algorithms represented in event processing (EP) languages for real-time analysis of the data streams. To prove the feasibility of the proposed framework, we implemented the system using a case study scenario of drought prediction and forecasting based on the Effective Drought Index (EDI) model. Firstly, we transform the predictive model into a form that could be executed by the streaming engine for real-time computing. Secondly, the model is applied to the ingested data streams and datasets to predict drought through persistent querying of the infinite streams to detect anomalies. As a conclusion of this study, a performance evaluation of the distributed stream processing middleware infrastructure is calculated to determine the real-time effectiveness of the framework.

## 1. Introduction

The emergence of the Internet of Things (IoT) has enabled the adoption and development of several real-time monitoring systems for diverse spheres of life such as energy management, health, smart environment, manufacturing, and security. As a result, the global IoT market is expected to hit over $10 trillion in 2025 [1]. For instance, in the environmental management and monitoring domain, ubiquitous sensors, actuators, instruments now provide real-time data acquisition, data-logging with telemetry capabilities [2]. These devices keep generating an avalanche of unbounded data streams related to the current status of the deployed environment. The enormous amount of data generated represents big data, which has the potential to provide more meaningful insight towards the timely understanding of complex environmental phenomena if properly analysed in real-time [3,4,5,6,7].

However, despite these potential benefits, building a real-time data analytics system is still challenging due to the variety of data, higher speed of data generation, volume of data to be processed, and the lack of a reliable, scalable and interactive platform [8,9,10]. In addition, processing and analysing of data for vital environmental information in real-time emergency cases are rarely done due to low adoption of state-of-the-art technologies [11]. Then, the application of big data technologies such as stream processing in the field of environmental monitoring would, therefore, be beneficial for predicting complex environmental phenomena for an effective decision-making process [12]. It allows scientists and researchers to integrate and analyse heterogeneous data from multiple non-interoperable sources in a stringent way [2,3,4,6,13]. Stream processing of data streams ensures enhanced analytic functionality, which would provide the necessary meaningful insight from IoT data and increases the productivity of processes for real-time data utilisation [3,6,12].

Hence, for multiple sensor nodes and large clusters, Apache Storm [14], Apache Flink [15], Apache *Kafka* [16], Apache Spark [17] are some modern stream processing architecture widely used for real-time data analytics. These technologies are mostly implemented in a cloud-based environment such as AWS EC2 [18], Microsoft Azure [19] or Google Cloud [20], for seamless integration with other IoT systems and platforms. However, its application in the environmental monitoring domain is limited due to the continued use of legacy devices within the infrastructure ecosystem, preventing integration and scalability. Therefore, the real-time processing of heterogeneous datasets using the big data platforms is not currently ideal due to the heterogeneity of data and higher communication latency caused by incompatible systems [10]. To use stream processing techniques effectively for real-time analysis of environmental data, a distributed framework is required to foster integration between heterogeneous data types and ensure system interoperability.

In this paper, as a solution, the authors introduce a stream processing middleware framework, called ESTemd, suitable for real-time event analysis of environmental management and monitoring data from heterogeneous systems using big data techniques. The use of established big data techniques would provide real-time analysis of the data, foster data integration, low-latency processing with high throughput [3]. We designed the ESTemd framework on top of publish/subscribe service strata. Hence, the presented framework solution is based on open-source Apache *Kafka*—a messaging system with robust data integration libraries (*Kafka* Connect) and stream processing API (*Kafka* Streams) to meet the need of real-time data/message processing in Confluent Platform [21]. Multiple data generated by heterogeneous producers’ nodes were ingested using Apache *Kafka* Connect APIs; transformed, and processed by the *Kafka* stream processing engine based on a chosen numerical model. Apache *Kafka* aims to unify offline and online processing by providing a mechanism for the integration of heterogeneous dataset as well as the ability to analyse and process streaming data over a cluster of machines [22].

Specifically, this paper makes the following contributions:

- It presents a distributed stream processing middleware framework for real-time analysis of heterogeneous data sources based on open source big data analysis techniques.
- Each component of the stream processing framework was presented in a layered level to emphasise the unified data pipeline.
- The presented framework is implemented in an environmental management and monitoring domain to demonstrate the effectiveness and adaptability of the proposed framework.

The rest of this paper is organised as follows: Section 2 motivates our work with examples relating to the current bottleneck in the domain requiring real-time processing. The background information on the research and related work were presented in Section 3, whereas Section 4 presents the distributed stream processing framework design. Then, we describe the case study experiment conducted to confirm the feasibility of the proposed framework in Section 4. This is followed by results and discussion of the performance of the proposed system in Section 6. Lastly, Section 7 concludes this article with some remarks and discussion of future work.

## 2. Motivation Goal and Scenario

The whole idea of a distributed framework is to facilitate interoperability between several heterogeneous components [23] and to hide the complexities of the underlying platform using a unified data pipeline to eliminate data heterogeneity. The framework layers can be categorised as a middleware which is a software layer composed of a set of sub-layers interposed between the application layer and different types of physical layers [23,24,25]. This ensures the ease of integrating heterogeneous devices while supporting interoperability within the diverse applications and services [26,27], which will be beneficial in any domain that deals with heterogeneous data sources.

Our goal was to develop a distributed stream processing framework for the analysis of heterogeneous data in the environmental monitoring domain using big data techniques. This approach will eliminate over-reliance on batch processing, slow processing period and data heterogeneity. To illustrate the current challenges, consider the following two examples. These two examples illustrate how heterogeneity of data and lack of an integrated system for real-time data access is affecting the application of big data techniques for real-time data analysis.

**Example** **1** (TAHMO)**.** *The Trans-African Hydro-Meteorological Observatory (TAHMO) (https://tahmo.org/) is a meteorological service with a vast network of weather stations on the African continent. This service provides current and historical environmental data from a dense network of hydro-meteorological weather monitoring stations located in several regions on the continent as a solution to bridge the huge data gaps in Africa. The weather stations network provides accurate localised weather dataset crucial for environmental monitoring and prediction.*

*A researcher could request access and download TAHMO raw data containing environmental data such as temperature, precipitation, relative humidity, wind direction, wind speed through the TAHMO web data repository (https://portal.tahmo.org). The near-real-time data are saved using relational schema and provided in different file format for use in numerical computation, modeling or analysis. TAHMO, does not offer a readily open system that could easily integrate with current big data platforms. Also, the heterogeneity of the data makes the data incompatible with big data platforms.*

**Example** **2** (University of KwaZulu-Natal—UKZN Weather Station)**.** *Another key challenge for climate monitoring in Africa is the availability of historical data in manual written form on paper and not catalogued electronically. Some of the legacy weather stations are in remote regions where data are manually recorded by meteorological officers and essential for any environmental monitoring modelling process. To mitigate this, UKZN, under the uMngeni Resilience project, has installed an automated weather station with the capability to access sensor readings online in real-time (http://agromet.ukzn.ac.za:5355/index.html). However, this system and dataset are incompatible with current big data platforms, and any form of big data analysis approach is clearly impracticable.*

From the examples above, it is seen that the heterogeneity of the data prevents the application of big data techniques while the heterogeneity of systems prevents seamless integration with current big data technologies, such as Apache Flink [15], Apache Storm [14] and Apache *Kafka* [16]. A framework with a unified data pipeline—serving as inputs for the big data platform will ensure streamlined data processing, synchronization, real-time analysis with high throughput is necessary. The proposed framework will ensure execution of a stream processing technique based on appropriate numerical model logic or algorithm irrespective of the data source and data type. The framework will also enable data integration and interoperability of distributed applications that run on different platforms for performance improvement.

## 3. Related Work

### 3.1. Background

In this section, we introduce the background information related to the proposed framework, including a description of the technological components, services, top-level interface with the big data techniques and platforms to be implemented. The distributed framework acts like a bond joining heterogeneous systems over heterogeneous interfaces for the application of stream analysis on the data using big data platforms. Moreover, through the use of the framework, data are sourced from a collection of apparatus forming the Data Acquisition Functional Group (FG), transformed and channelled via a unified data pipeline for utilisation in the big data infrastructure. The results of the analysis are published through the Data Publishing FG.

**Figure 1:**
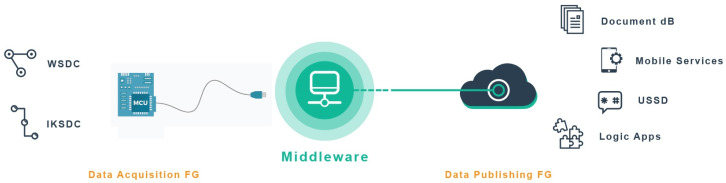
Overview of the distributed system.

Figure 1 shows an overview of the framework as a distributed three-tier system architecture that stretches across multiple systems or applications. The proposed distributed framework consists of the following sub-systems: Data Acquisition FG, Middleware, and the Data Publishing FG. The data acquisition components are responsible for the acquisition of data from heterogeneous sources using APIs. These sources have different specifications and characteristics which cannot be generalised. The middleware ingests the data using compatible APIs before the data are processed by the middleware’s streaming engines. On the other hand, the output results obtained by way of real-time analysis of the data streams are published by the data publishing FG components through designated APIs. The authors believe this approach simplifies the integration of heterogeneous data types from multiple sources for analysis by the big data platform.

In the middleware component is a stream processing engine that executes computational models and logics written in Event Processing Language (EPL) for real-time processing of the data streams. The processing engine provides high-level programming models such as EPL with built-in functions for event filtering, correlation, and abstraction (e.g., Flink, Storm, Kafka, Samza). The EPL is used to define patterns, prediction model logics—specifying the parameter threshold, formula—including temporal relations, aggregations, indices models and event correlations. The event patterns used in the inference mechanism are classified by [28] as (i) selection pattern—used to detect a simple event; (ii) windows data—which is used to assign windows of stream of data for scope restriction; (iii) temporal sequencing of events—used to specify temporal ordering of events; (iv) pattern combinations—for combining several patterns using logical operators (AND, OR, NOR etc.) with temporal connectors (while or until).

#### 3.1.1. Stream Processing Engines and Platforms

Stream processing as a subset of event processing is the type of computing which captures the occurrence of real-world incidents in the form of time-series observation and processes the data in real-time. The processing involves the use of an EP engine that performs computational operations such as averaging, pattern matching, event prediction (forecasting), event filtering, correlation, and abstraction on the input data streams or dataset. The information from the mined data is consumed by custom application or information systems. Over the years’ various stream computing platforms have been developed [29], some are developed following a publish/subscribe pattern with an integrated EP engine and message broker. In contrast, others are standalone EP engine that requires an external resource (e.g., message broker). Apache Storm [14], Apache Flink [15], Apache *Kafka* [16], Apache Spark [17], IBM InfoSphere Streams [30], DataTorrent Real-time Streaming (RTS), Apache Samza [31], SQLStream s-Server, Apache Storm [14], etc. are some notable examples for event processing engines and platforms. A summary of features of some existing stream processing engines, platforms and message brokers is shown in Table 1.

Table 1 highlights the salient features of different stream processing engines, platforms and message brokers. This table serves as a guide outlining the features of Apache *Kafka* as a suitable processing architecture for this case study. In the feature summary table, each specific details are highlighted, if the detail of a particular feature is not known we keep the cell in empty status. From the available open-source architectures, *Kafka* provides critical features in terms of system scalability, high availability, high performance (up to 100,000 message/s on a single server) and persistent querying of the input streams in real-time. These features are essential in processing heterogeneous data streams in the environmental monitoring and management domain. Features such as EP engine, integrated message broker, SQL-like querying and the ability for centralised cluster management are also supported by *Kafka*. A detailed benchmarking and comparison is available in [32,33,34,35].

#### 3.1.2. Apache Kafka

**Figure 2:**
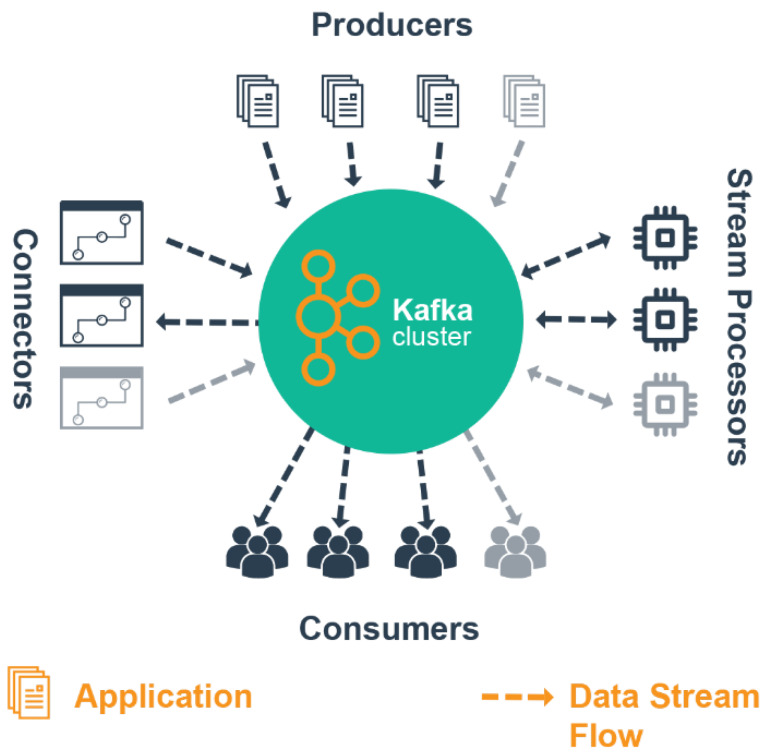
A simple Apache Kafka ecosystem [16].

Apache *Kafka* is an open-source distributed event streaming processing engine by Apache [16]—available in the form of IaaS (Infrastructure-as-a-Service) and used to provide the functionality of analysing data streams using filters, aggregations, joins on a set of window data based on different predefined patterns for predictive inference generation. It is a streaming platform with the capabilities to publish and subscribe to data streams, store streams of data in a fault-tolerant way and process the data streams in real-time [16]. The streaming processing engine processes sensor data streams or datasets in real-time to determine event patterns from incoming sensors’ observation/readings and correlate the data with a predefined/preset value threshold for predictive analysis. The platform is similar to an enterprise messaging system based on the ability to process data streams from the heterogeneous producers using APIs in a fault-tolerant way as they occur in a producer-publish and consumer-subscribe fashion (Figure 2).

**Figure 3:**
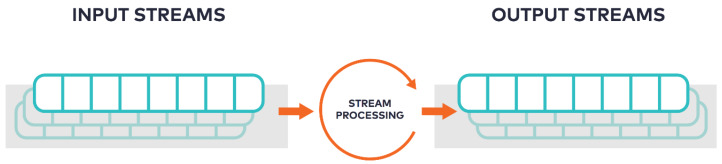
Infinite flow of streams [16].

Apache *Kafka* processes and analyses streaming data through *Kafka*-centred data pipelines that reliably get data between heterogeneous systems or applications with core *Kafka* Connect APIs. *Kafka* also utilises KSQL—a streaming SQL engine for *Kafka* to perform real-time persistent querying without the need to commit the data stream to the database like conventional extract, transform, load (ETL) systems [36]. Data streams which are never-ending potential flow of data records and the input streams are analysed as generated through the stream processing engine to produce the output streams (Figure 3). Several use cases in the literature have shown the significance of real-time predictive analysis from a stream of sensors data or compatible data sets for smart cities, healthcare, energy management, to business intelligence [37,38]. This provides a huge benefit in IoT-enabled environmental management and monitoring systems for real-time monitoring of complex environmental phenomenon, which is crucial for predictive analysis.

Apache *Kafka* can be run as a cluster on a local server or implemented in a cloud environment, with every producer connected to the cluster using the *Kafka* Connector APIs, processed by the stream processors. The cluster stores a stream of data records in categories called topics. Each record consists of a key, a value, and a timestamp. The *Kafka* stream processing engine consumes the input stream from the available topics, producing an output stream to one or more output topics, effectively transforming the input streams to output streams.

The application use case of Apache *Kafka* in the context of environmental monitoring and management is to serve as a centralised messaging system that facilitates exchanges of messages between heterogeneous devices and systems. The stream processing of the heterogeneous data is performed using the *Kafka* streams API in real-time. This will facilitate the decoupling of data and system dependencies to ensure seamless integration and utilisation of existing legacy systems with newer systems for integration with big data platforms.

#### 3.1.3. Apache Kafka Features

##### Topics

In a *Kafka* cluster, messages are categorised to topics based on similar attributes. Each topic is similar to tables in a database such as PostgreSQL—however, without the constraints. In *Kafka*, several topics can be created to categorise the similarity, with each topic uniquely identified by its name. Topics are split into partitions in an ordered list with messages/data in each partition of the topic identified using an incremental identifier called an offset [39]. The messages or event records generated from the Producer are written to the appropriate topic in chronological order or sequence of arrival, where consumers make use of the messages by reading the messages from the topics.

##### Brokers

A *Kafka* cluster is composed of multiple brokers with each brokers acting as a server in the cluster. The brokers contain the topic partitions and are uniquely identified with an integer ID. Each broker contains topic partitions with topics’ data in a distributed form. Partitions are one way in which *Kafka* enables horizontal scalability for replication of messages across cluster [40]. Replication is a critical criterion in any distributed systems, hence, *Kafka* ensures a replicated copy of all topics is available with another broker within the cluster depending on the topic replication factor.

##### Producers

The producers in *Kafka* are the data/message sources that write to topics, which are subsequently written to partitions and saved on the brokers in the cluster. They are the event producers and vary from sensors, devices, manual or automatic systems.

##### Consumers

The consumers ingest or consume the messages/data generated by the producers by subscribing to a particular topic [41]. The consumers are automatically configured and programmed with read access to connect with the appropriate broker to read the streaming data. In the case of broker failure in the cluster, consumers automatically fetch the data from the next available broker in the cluster. Data/messages in the partitions are consumed in the order based on the offsets. Consumers offsets are like standard bookmarks that enable the consumers to know where to start reading the streaming data of the topic. In cases where there is more than one partition of a topic, the data/messages are read in parallel by the consumer and randomly from multiple partitions for the same topic.

##### KSQL

KSQL is an abstraction of the *Kafka* Stream API that is similar to the SQL with almost identical syntax and mode of operations to normal SQL and allows the continuous queries on infinite streams of data [39]. KSQL allows the stream processing of data streams using SQL-like operators such as WHERE clause for data filtering and transformation, JOINS for data enrichment, and data manipulation with scalar functions. KSQL consumes streams of data mostly dataset in AVRO, JSON or CSV stored in *Kafka* topics and processes them using SQL-like queries and the output are stored in a *Kafka* topic.

#### 3.1.4. Confluent

The confluent platform is an enterprise streaming platform based on open-source Apache *Kafka* developed to achieve a fully streaming architecture in the IoT domain for different streaming pipelines of different sensor types and devices [21]. The central platform provides the ability to configure various data pipelines for data integration and interoperability. The confluent dashboard accessible through the localhost provides an integrated platform to monitor the health of the clusters; brokers in the cluster, system load measurements and performance view with the aggregate statistics at a broker or topic level.

### 3.2. Related Research on the Application of Big Data Analytics

Big data analytics has become an integral research area with the need to figure out how to harness and gain actionable insights from big data [42]. The enormous datasets generated on daily basis from avalanche of devices and systems have exceeded the capacity of commonly utilised analysis techniques and devices to catch, process, and correlate information inside an allowable bearable period. Current methods such as machine learning, data mining have been used to analyse and extract useful information from enormous digital data generated from a plethora of systems and devices.

Recently, climate change has been fueling the need for more crucial data for predicting and forecasting the environment. This has led to the application of cutting-edge technology for data acquisition in conjunction with the use of existing legacy systems datasets. The data generated from these infrastructures are huge and increases dramatically. Therefore, the application of big data analytics technologies is essential for building an efficient real-time processing system for the environmental management and monitoring domain [9]. In the past, ETL techniques with Relational Database Management Systems (RDBMS) are used due to the homogenised nature of the data and relatively small volume. Currently, RDBMS cannot be used due to the enormous size of the data, the complexity and heterogeneity of the data.

Big data analytics has been studied in many systems such as tsunami detection systems and early warning systems in order to increase the efficiency of processing massive datasets [43]. Most of these analytics solutions are focused on using batch processing techniques and technologies like the Hadoop, for the batch processing of large data sets across distributed clusters using the MapReduce programming model [44]. However, considering the advantages of big data analytics and technologies, the application rate in the smart environment and environmental monitoring domain is slow due to underlying fundamental challenges of data and systems heterogeneity [45,46]. While a comprehensive review of existing application scenarios is beyond the scope of this paper, some notable examples are discussed and investigated.

In Ref. [47], the Real-time Observatories, Applications, and Data-Management Network (ROADNet) project was the successor to the Antelope Environmental Monitoring System developed for large-scale real-time seismic monitoring. The ROADNet project aims to develop an integrated, seamless environmental information network to process and analyse geophysical, ecological and physical data in real-time through system interoperability [47,48]. As in our framework, it utilises third-party open-source software, in this case for visualisation (based on Keyhole Markup Language (KML)). However, it does not address seamless data integration issues from heterogeneous devices.

Real-time Environment for Analytical Processing (REAP) is a cyber-infrastructure development project to access, monitor, analyse and present information from field-deployed sensor networks, for both the oceanic and terrestrial environments and across multiple spatio-temporal scales [49,50]. However, it is a near real-time analytic processing framework for executing scientific models for data streams from the sensor network and does not integrate data from other sources. Eleftherakis and others [51] also proposed a distributed sensor network architecture for IoT using middleware for messages dissemination. The proposed middleware facilitates interacting between things without further processing. Also, [52] apply ontological concepts and semantic stream processing technologies to facilitate combination, comparison, and visualization of heterogeneous data from various sources using C-SPARQL [53] as the stream processor, and reasoning capabilities through SPARQL extensions [54].

Furthermore, there have been several evaluation studies for resource optimization and utilisation [35,55,56,57]. In Ref. [55], the authors address the scheduling of big data services in cloud-based environments as a means to minimize the amount of utilised resources. To do so, they design a trust aware scheduling solution called BigTrustScheduling that consists of three stages: VMs’ trust level computation, tasks priority level determination, and trust-aware scheduling. They experiment with a real Hadoop cluster environment using real-world datasets. Their approach is meaningful when scaling up the real-time processing of big data to increase the performance of big data services execution. The authors of [56] propose a system to support intent-based multi-tenancy in modern distributed stream processing systems. The system allows each job to specify critical requirements that capture latency and throughput needs towards maximizing the system utility. This is possible with the retention configuration of the messages in the broker. In Ref. [35], the authors investigates the impact of processing time on the number of stream records of the streaming engine towards improving the processing efficiency. The experimental result shows a higher duration of time interval causes rapidly processing speed. The challenge identified from the study has been mitigated by *Kafka* through the use of dedicated topics with offsets.

The aforementioned related works focus on the application of event processing techniques, resource optimization with efficiency, which are relevant in the context of this research. To tackle some limitations and harness the benefits of the discussed works, we propose in this paper, a distributed solution that employs stream processing techniques, resource optimization and multi-tenancy approach for real-time processing of heterogeneous data sources in the environmental monitoring and management domain.

## 4. Distributed Stream Processing Framework Design

### 4.1. High-Level Architecture

**Figure 4:**
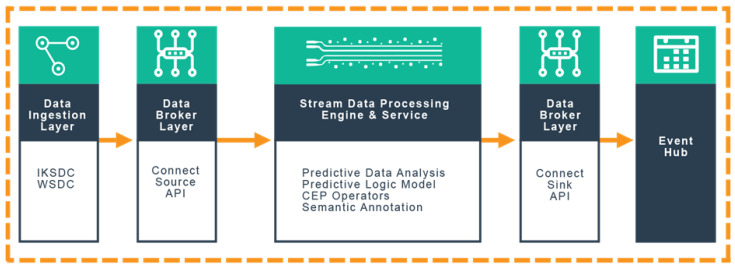
The high-level view of ESTemd distributed middleware framework for real-time analysis of environmental management and monitoring data.

The proposed distributed stream processing framework utilised Apache *Kafka* and the *Kafka* streaming processing engine in an enterprise-based Confluent environment. The high-level architecture of the framework is depicted in Figure 4 and categorised into five layers: (i) data ingestion layer, (ii) data broker layer, (iii) stream data processing engine and services, (iv) data broker layer, and (v) event hub. The framework will process real-time data ingested from connected devices for real-time data analytics. Firstly, heterogeneous devices (producers) which form the data ingestion layer, acquire data from sensing devices, automated fixed station and manual weather stations. The data stream generated is captured by the *Kafka* Connect Source API to respective topics based on the data properties and attributes [22]. The topics are processed and analysed in real-time by the *Kafka* streaming processing engine using appropriate numerical model logic represented in EPL.

**Figure 5:**
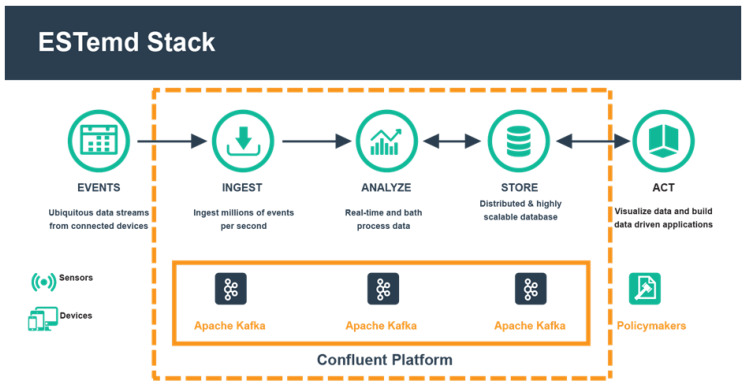
ESTemd Unified Middleware Framework Stack.

The stream processed outputs (data) are passed to the Event hub layer for storage into an appropriate storage medium using compatible *Kafka* Connect Sink API. This layer consists of necessary APIs that make the data materialize for elastic search indexes for visual representation analysis in the form of charts, lines and tables by Kibana. The output data is interpreted by the policymakers or further consumed by another set of consumers or systems connected to the middleware. Figure 5 below presents the ESTemd framework as a stack illustrating the data flow.

### 4.2. ESTemd Framework Layers

#### 4.2.1. Data Ingestion Layer

**Figure 6:**
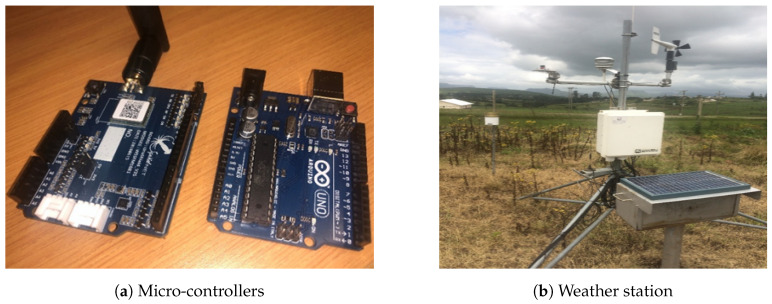
IoT devices and automated weather station.

In the ESTemd framework, the data ingestion layer is a component of the Data Acquisition FG and consists of sensors, weather stations, legacy system, or databases—called Producers (Figure 6). The vast range of heterogeneous devices produces the time-series data in different data formats. The generated data include not only streaming data but also data from legacy systems and stored datasets. The data after ingestion is transformed by the *Kafka* source connectors API. The *Kafka* source connectors API is defined in accordance with the producer’s data format. It buffers the incoming data streams from the producers and transfers the data to the broker. Furthermore, It helps to achieve better fault tolerance with load balancing in the eventuality of component failure.

#### 4.2.2. Data Broker Layer (Source)

**Figure 7:**
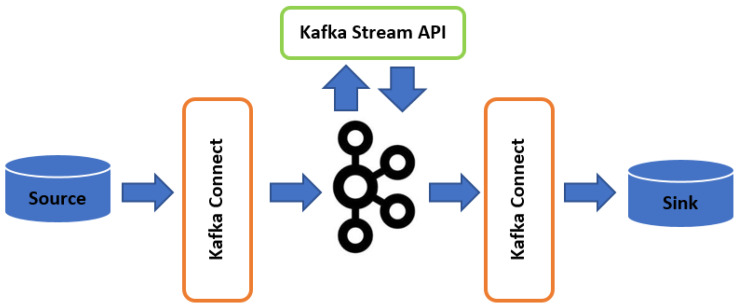
Overview of the Apache *Kafka* streaming engine [16].

The data broker layer performs the coordinated processing and transformation of the unbounded data stream coming from various heterogeneous devices and systems in the data ingestion layer with multiple transport protocol support. The data are received from the Data Ingestion layer using *Kafka* Connect Source API, with additional data preprocessing performed. Apache *Kafka* Connect Source API acting as a broker will buffer the incoming data streams from the producers and help to achieve better fault tolerance and load balancing in the eventuality of component failure [22,58] as depicted in Figure 7 below.

*Kafka* Connect Source provides the set of API classes based on different messaging protocols to facilitate stream messages from different producers’ gateways channels to the *Kafka* cluster. The *Kafka* Source Connectors broker buffers the incoming messages, keeping it in a queue and are replicated across all the brokers in the cluster [22]. The connectors automatically perform data transformations on the messages to make it easier to process. The source connectors ingest the data streams table or entire database and pass it on to the appropriate *Kafka* topics in the broker.

Typically, a *Kafka* source connector ingests entire databases and streams messages from the producers’ gateway channels to respective *Kafka* topics in the cluster. The *Kafka* Source Single Message Transform makes real-time light-weight modifications to the raw messages before publishing to *Kafka* streaming engine [22,59]. There are several source connectors available on the *Kafka* platform, depending on the native language of event producers. For example, *Kafka* Connect MQTT, *Kafka* Connect RabbitMQ, *Kafka* Connect JDBC, *Kafka* Connect CDC Microsoft, RabbitMQ, HDFS, HTTP, MongoDB, Neo4j, Cassandra.

**Figure 8:**
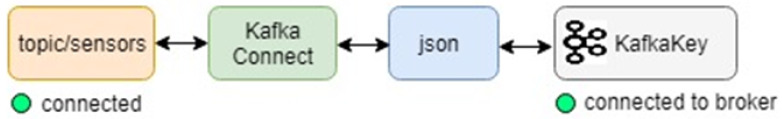
Node-Kafka-broker data pipeline programming flow [60].

The programming flow of data is such that the data from the first node are fetched from the sensing devices encoded in a simple JSON format using *Kafka* Connect API before being transmitted to other nodes, as illustrated in Figure 8 above. The messages represented in the JSON-LD are transmitted to the next layer *node-red-contrib-Kafka-node* [60]. The Apache *Kafka* broker in the cluster hosts some topics for aggregating similar data attributes. This layer is highly scalable using a publish-subscribe event bus which ensures that heterogeneous data streams are captured with minimal loss.

#### 4.2.3. Stream Data Processing Engine and Service Layer

The goal of the stream data processing layer is the stream processing of the transformed stream data collected by the *Kafka* broker. The stream of data flowing through several *Kafka* topics in *Kafka* broker is processed using *Kafka* stream APIs to detect events in the time-series data streams (Figure 7). This layer further consists of the predictive data analytics model, represented using EPL—in the form of *Kafka* CEP operators—used to transform the model into a logical structure that can be implemented by the *Kafka* stream API.

##### Predictive Data Analytics

This is the data and processing analytics components of the stream data processing and service layer—used to perform several analytics functionalities. The streaming dataset is queried with SQL-like operators based on the appropriate model to gain predictive insights.

##### Kafka CEP Operators

A stream processing engine utilises the use the CEP operators to identify meaningful patterns, relationships and gain predictive insights from streams of an unbounded dataset. *Kafka* streaming processing engine primitive operators such as Filter (), Map (), FlatMap (), Aggregation (), Projection (), Negation () are used for various combinations and permutations of parameters of the streaming data [44]. These operations are invoked on the *Kafka* topics in the cluster(s) using KSQL. Once a pattern(s) is/are identified and extracted, the KSQL will encapsulate it into a composite (derived) event to be published into an output *Kafka* topic saved in the cluster or the form of a message to a secondary index by the event publishers.

#### 4.2.4. Data Broker Layer (Sink)

*Kafka* Sink Connector streams the data out of *Kafka* clusters to other secondary indexes such as Elasticsearch or Cassandra using *Kafka* Source Single Message Transform to make lightweight modifications to *Kafka* messages before writing the output to an external repository. The stream processed outputs are delivered from the *Kafka* topics to the secondary indexes for visual representation and analysis with Kibana [61] or offline batch analysis with Hadoop. In the context of this research, the output data will be consumed and used by policymakers as a critical output of the middleware. The relevant examples of *Kafka* Sink Connectors APIs are *Kafka* Connect Neo4j, and *Kafka* Connect HDFS, *Kafka* Connect HTTP, *Kafka* Connect for MQTT-JSON.

#### 4.2.5. Event Hub

The output from the stream processing engine is stored in the respective output topics and can be further saved to a data sink. The data sink in the event hub acts as a buffer to save the output topic data from the streaming engine. The output topic data can be saved to secondary indexes such as MongoDB [62], Cassandra [63], NoSQL databases for an offline longer time series analysis or immediate visual analysis using AKKA [64], KIBANA [61] or Apache Zeppelin [65] to gain further insights.

## 5. Use Case

This section presents the implementation of the ESTemd framework for real-time analysis of environmental management and monitoring data. This use case scenario is based on the application of the stream processing engine towards real-time prediction or forecasting of drought from heterogeneous data sources using EDI model. In order to tackle environmental issues, very different types of models need to be combined, represented in EP language before implementation. The data sources for this implementation are derived from heterogeneous sources.

### 5.1. Experimental Setup

All experiments described in the following sections have been performed using hardware provided by the Unit for Research and Informatics for Drought in Africa (URIDA), Centre of Sustainable Smart Cities (CSSC), Central University of Technology, Free State, South Africa. The entire stream processing cluster and infrastructure could be deployed in some cases as docker containers and managed by kebenetics in cloud infrastructure such as AWS, Virtual Machine (VM). The cloud services such as AWS provides computing resources to run instances types with combination of CPU, memory, and disk options for appropriate performance. Also, bare-metal computer or local servers could be utilised depending on the requirement and scale of the ecosystem. In this research, for implementation, a single node cluster was created on a physical machine—Apple Macbook Pro with Intel Core i7 3.1GHz Quad-Core processor running macOS Mojave; the VM is running Ubuntu Linux with Intel Core-based processor as a base machine of the distributed middleware module.

The infrastructure is composed of two clusters: (1) a cluster running on a local machine with a Quad-core Intel CPU and 16GB RAM hosts the ZooKeeper, an instance of *Kafka* broker, an active controller and *Kafka* broker; (2) *Kafka* client hosting the *Kafka* streaming engine API and the KSQL for persistent querying of the streams in real-time, both clusters monitored and managed through the Confluent streaming platform. The strings of commands below are used for starting an instance of Confluent streaming platform through the terminal.

**Commands for starting the cluster through a Terminal**

Open Terminal (Ctrl+Alt+T)

cd user/location/Confluent/confluent-5.2.1/bin/confluent

user/location/Confluent/confluent-5.2.1/bin/confluent Start

Zookeeper, Kafka, Schema-registry, Kafka-rest, Kafka Connect, Ksql-server UP

Control-center UP

**Figure 9:**
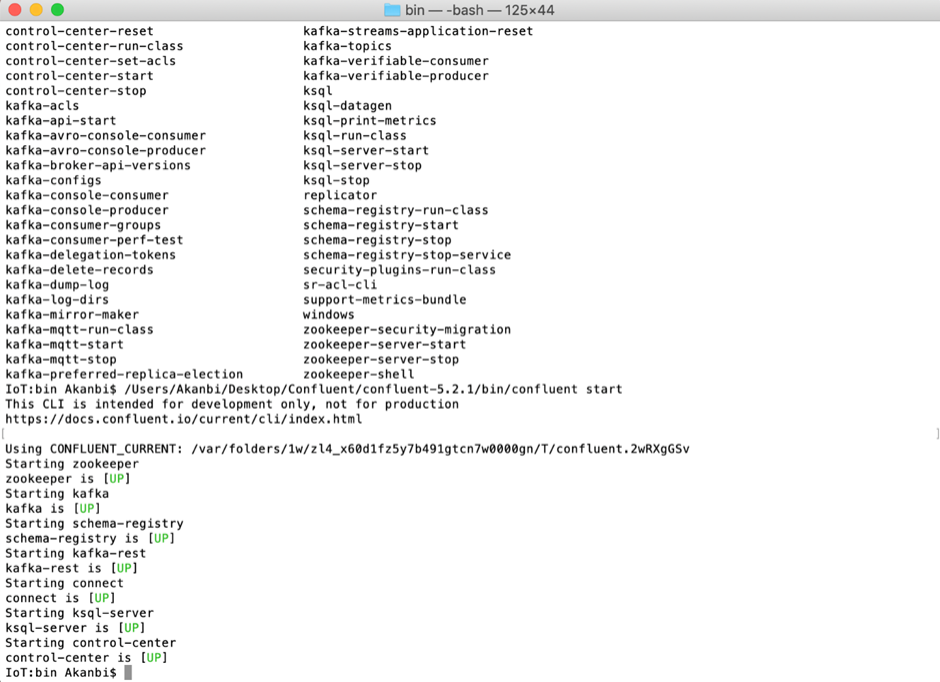
Starting Confluent Platform in the Terminal.

The Confluent Platform is started through the Terminal by invoking the bash file to launch an array of services such as zookeeper, *Kafka*, schema-registry, *Kafka*-rest, *Kafka* connect, KSQL-server and the control-center services all in a sequence. Figure 9 below depicts starting the *Kafka* broker through the Command Line Interface (CLI) on the local server. By executing the configuration files, the Confluent streaming platform is started. The platform control center interface can be accessed on the localhost server through a web browser by going to http://localhost:9021/. After the execution is completed, the four main API—producer API, consumer API, Connector API and Streams API are used to interact with the *Kafka* broker through the control center. The platform control center interface provides an integrated approach to monitor the health of the clusters, brokers, topics, measure the system load, performance operations and even aggregated statistics at a broker or topic level. Confluent Platform provides a broker-centric view of the clusters, used to perform end-to-end stream monitoring, configure the data pipeline using *Kafka* Connect and query the data streams, also with the ability to inspect streams, measure latency and throughput. Furthermore, from the experiment, we study the performance of our solution in terms of latency and execution response time. This is an essential factor in evaluating the effectiveness of the proposed solution.

### 5.2. Data Sources

To implement the proposed model for this research, two datasets have been used. Sensor data from a WSN and the dataset from a weather station at a constant stipulated interval are fed into the system for drought prediction using the EDI model. Each reading entry is in the form of a key-value pair containing the information and the time when data was collected for the stream processing.

### 5.3. Predictive Model Logic—Effective Drought Indices (EDI)

EDI has been identified as a good index for determining and monitoring of meteorological drought and categorizes the severity of a drought event on a scale [66]. The analysis of the streaming data will be based on the EDI model. The EDI model is represented in the form of a logic using the EP language. The datasets would be processed and analysed based on the EDI for determining and profiling droughts in real-time on a daily using the *Kafka* streaming engine. The EDI formula set, where precipitation is recorded, is presented below:

(1) $$EP_{n}=\sum_{n-1}^{i}\left[\frac{\left(\sum_{m-1}^{n}Pm\right)}{n}\right]$$

where, *EPn* represents the valid accumulations of precipitation of each day, accumulated for *n* days, and *Pm* is the precipitation for *m* days, m=n. In Equation (1), if m/n = 365, then, EP becomes the valid accumulation of precipitation for 365 days divided by 365.

(2) $$DEP_{n}=EP_{n}-MEP_{n}$$

$DEP_{n}$ in Equation (3) represents a deviation of $EP_{n}$ from the mean of $EP_{n}$ (MEP)—typically 30-year average of the EP.

(3) $$EDI_{n}=DEP_{n}/SD\left(DEP_{n}\right)$$

$EDI_{n}$ in Equation (3) represents the Effective Drought Index, calculated by dividing the DEP by the standard deviation of DEP-SD (*DEPn*) for the specified period. In order to detect the onset of drought based on the EDI prediction model, analysis and manipulation were performed on the datasets using Kafka operators—Filter (), Map (), FlatMap (), Aggregation (), Sum (), Average () used to represent the EDI model in KSQL [22]. The data streams in the *Kafka* topics are queried in real-time using the EDI model in KSQL. The dataset containing the historical precipitation values will be read from a file to a *Kafka* topic. The output of the persistent query is committed to the output *Kafka* topic in the form of drought index value belonging to one of the four category of the EDI.

The drought levels are categorised into four classes in EDI (Table 2) [66,67]. After computation using Equations (1)–(3), the output value of the EDI which ranges from negative to positive determines the category of the drought, which indicates the intensity of the drought, giving a clear definition of the onset, end and duration of the drought. For example, a value of −1.05 indicates near-normal drought. The interpretation and classification of the drought based on the output values of the EDI calculation are published by the event hub.

### 5.4. Methods

**Figure 10:**
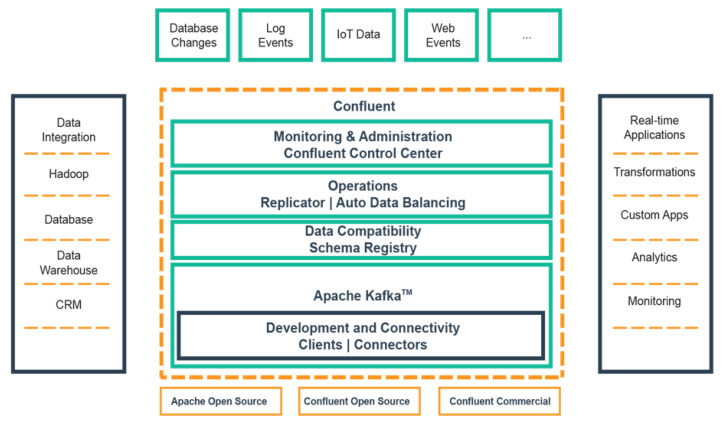
The Confluent Enterprise Streaming Framework [16].

To achieve a fully streaming processing architecture for the heterogeneous data using Confluent. The platform provides the ability to configure, monitor and manage the data pipelines of producers using a variety of several connectors APIs for different native clients and processes the input streams in real-time using Confluent KSQL (Figure 10). The time-series environmental monitoring data from deployed sensors and datasets are ingested by the broker and saved to designated *Kafka* topics. Several producers can write messages records to the same topic. In this instance, the messages to be processed are read from the appropriate *Kafka* topic and executed based on the numerical computational model with the output record saved to the output *Kafka* topic. The output messages are consumed from the designated output topic by several consumers in the form of custom applications or target systems. In the same way, multiple consumers forming the consumer group will subscribe to the output topic with each consumer in the group consuming the output messages from a different subset of the partitions of the same topic as part of a multi-tenant solution.

**Figure 11:**
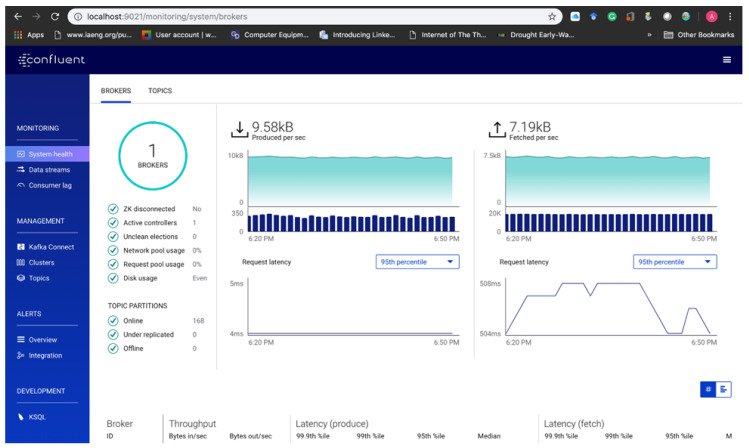
The Confluent platform interface.

Unique topics are created for each type of input data streams in the application. This allows the grouping of a particular type of sensor data in the same topic, and consumers can retrieve the right data through the sensor group. After starting Confluent, the streaming platform interface can be accessed through the localhost server on port 9021 (Figure 11). The dashboard provides an integrated approach to monitor the health of the clusters, brokers, topics, measure the system load, performance operations and even aggregated statistics at a broker or topic level. Confluent Platform provides a broker-centric view of the clusters, used to perform end-to-end stream monitoring, to configure the data pipeline using *Kafka* Connect and to query the data streams, also with the ability to inspect streams, measure latency and throughput.

#### 5.4.1. Configuring Unified Data Pipelines Using Kafka Connect

**Figure 12:**
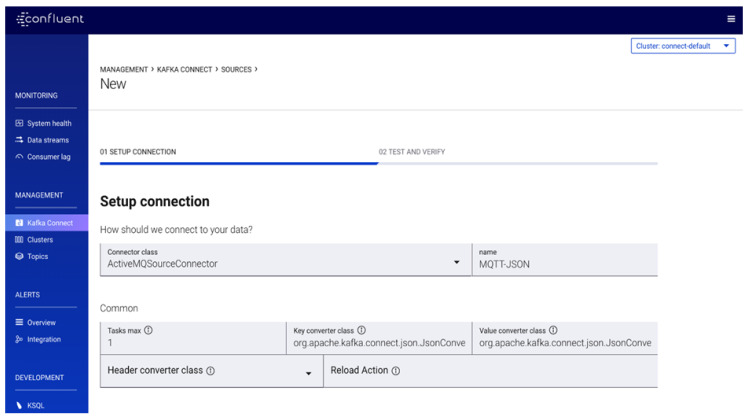
Configuration of Kafka Connect APIs in Confluent platform.

The Confluent Platform ensures the integration of all services and managing of the data connectors to connect data emanating from heterogeneous producers in one place. However, data can be pushed to the *Kafka* broker or pulled from it through the use of either traditional producer and consumer clients or using the Connect APIs. The advantage of using client’s APIs in production environment allows a custom application to be developed to directly push and pull data from the broker. On the other hand, Connect APIs are used for external datastore and provides features for parallelization, offset storage, support for different data types and REST APIs management. For this implementation, the integration of heterogeneous data sources is made possible through *Kafka* Connectors; it provides meaningful data abstractions to pull or push data to *Kafka* brokers [68]. *Kafka* connectors are forward and backward compatible with vast data representation formats such as XML, JSON, AVRO etc. The configuration of the *Kafka* connector is through the *Kafka* Connect management console (Figure 12). There are two major types of *Kafka* connectors—the *Kafka* Source Connector for connecting to the producers and the *Kafka* Sink Connector for connecting to the secondary data storage indexes [69]. In the *Kafka* Connect management console, the connector class, key converter class, value converter class are defined for the data formats for the *Kafka* Source Connector and the *Kafka* Sink Connector to achieve common serialization format and ecosystem compatibility. This will specify the *Kafka* messages and convert it based on the key-value pairs using *key.converter* and *value.converter* configuration settings. The entire data pipeline in the middleware infrastructure is represented in JSON. Hence, for JSON, the *key.converter* will be represented as “*key.converter*”: “*org.apache.kafka.connect.json.JsonConverter*”. If we want *Kafka* to include the schema we insert “*key.converter.schemas.enable=true*”. The same will be applicable for the *value.converter*. To ensure high availability for an increased number of tasks, two or more instances are defined and running.

#### 5.4.2. Producers Messages

**Figure 13:**
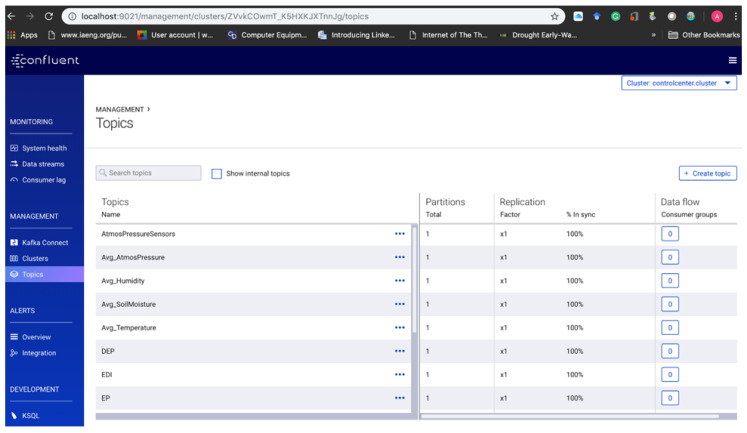
Topics created in the Kafka broker through the Confluent platform.

In this research, five unique topics are created to cater for and categorise the messages—temperature readings, humidity readings, atmospheric pressure readings, precipitation readings and the soil moisture readings from the producers. Table 3 below shows the type of readings and the respective topics created. New topics (Figure 13) were further created to store the output of the processed streams.

**Figure 14:**
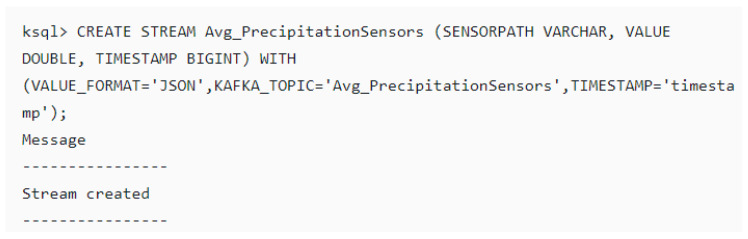
Creating topic in the Kafka broker through the terminal.

The manipulation of the *Kafka* topics messages using CEP operators based on the EDI model formula will yield new processed messages/data saved in the output topics. Furthermore, performing the average operator (Avg ()) on the existing input topics will create five (5) new additional output topics namely: Avg_Temperature; Avg_Humidity; Avg_AtmosPressure; Avg_SoilMoisture; Avg_PrecipitationSensors. Additionally, a further six (6) *Kafka* topics will be created to store the output of the EDI computations, namely: DEP, Standard deviation of DEP, EP, Mean of Effective Precipitation (MEP), Sum of Precipitation (Sum_Precipitation) and EDI. Lastly, a new topic that stores the historical precipitation data from the file—“HistoricalPrecipitation” will be created for calculating the MEP. Therefore, there are 17 *Kafka* topics in our broker, all created with the same number of partitions and replication factors across the cluster for high availability. Figure 13 and Figure 14 show the creation of the topics in the Confluent platform and KSQL code of how it was created in the terminal respectively.

#### 5.4.3. Workflows

The data streams generated by the producers are passed on to the *Kafka* topics in the *Kafka* broker for stream processing. The *Kafka* cluster is composed of two (2) nodes having similar settings running Intel-based processors. The *Kafka* broker runs operators and user-defined functions inside the JVM. The EDI computational process performed on the data streams using KSQL will generate new outputs that will be committed to the appropriate topics in the broker. KSQL performs persistent line queries, filtering and aggregation of data records based on the numerical computational model.

#### 5.4.4. Persistent Querying/Analysis of the Data Streams Using KSQL

**Figure 15:**
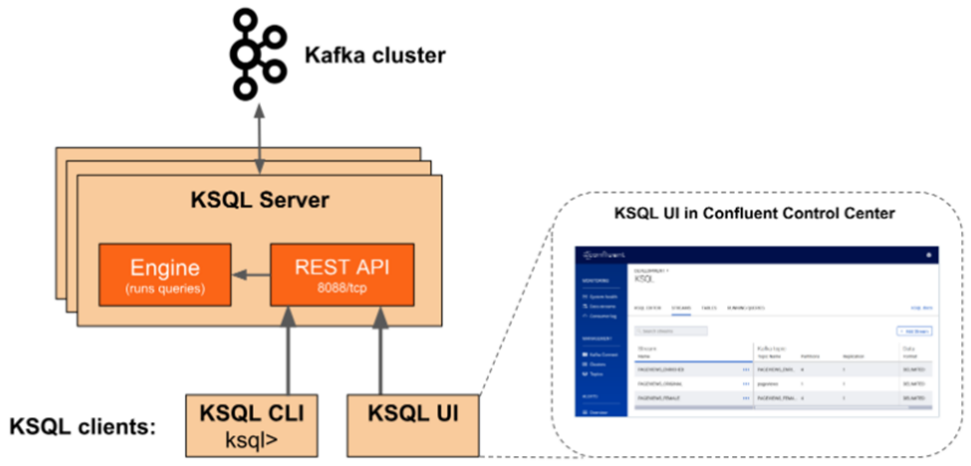
KSQL cluster interfacing with the Kafka broker [16].

Each record or message from a producer is typically represented as a key-value pair, and the streams of record are processed and analysed in real-time with the smallest amount of latency through the help of *Kafka*-SQL (KSQL). KSQL Server consists of the KSQL engine and the REST API. KSQL Server routines communicate with the *Kafka* cluster through the KSQL UI (Figure 15). The data analysis with stateful processing, aggregation and windowing operation for time-series analysis are executed.

**Figure 16:**
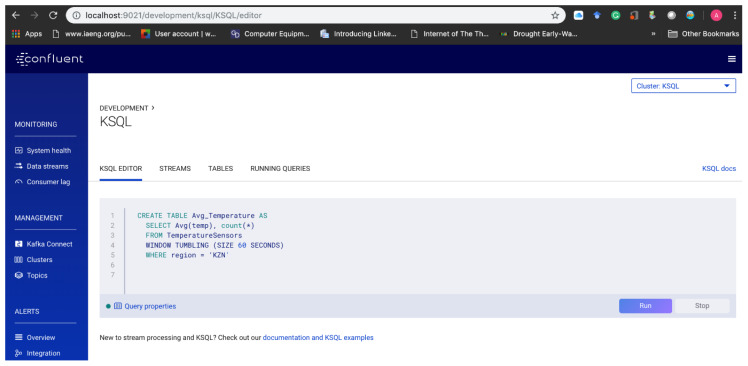
KSQL editor in the Confluent Platform.

KSQL consumes the data streams stored in *Kafka* topics—TemperatureSensors, HumiditySensors, AtmosPressureSensors and SoilMoistureSensors; which are mostly structured data set in JSON but could be in a format like AVRO or delimited formats (CSV) by using the appropriate *Kafka* Connect API for the data pipeline. Queries are performed through the use of KSQL cluster connected to the *Kafka* broker. KSQL persistently queries the infinite input streams through the appropriate topics to execute the EDI model logic (Equations (1)–(3)) transformed into the querying algorithm. The querying algorithm is programmed using the KSQL editor console (Figure 16) for the persistent querying of the topics.
**Algorithm 1:** KSQL Querying Algorithm The querying algorithm is a logic generated from the EDI model Formula (1)–(3): Generate KSQL (DStream) STEP (1) FOR historical precipitation dataset
 IF dataset is Filesystem WHERE file format is. xslv
 READ file (.csv)
 CREATE Table “HistoricalPrecipitation”
 SAVE file (.csv) to Table “HistoricalPrecipitation” (2) FOR Sum_Precipitation = SUM (PrecipitationSensors)
 CREATE Table “Sum_Precipitation”
 SAVE “Sum_Precipitation” to Table “Sum_Precipitation” (3) FOR EP = (Sum_Precipitation)/(Time Frame)
 CREATE Table “EP”
 SAVE “EP” values to Table “EP” (4) FOR MEP = Mean (HistoricalPrecipitation)
 CREATE Table “MEP”
 SAVE “MEP” values to Table “MEP” (5) FOR DEP = EP - MEP
 CREATE Table “DEP”
 SAVE “DEP” values to Table “DEP” (6) FOR SD(DEP) = Standard deviation (DEP)
 CREATE Table “SD(DEP)”
 SAVE “SD(DEP)” values to Table “SD(DEP)” (7) FOR EDI = DEP/(SD(DEP))
 CREATE Table “EDI”
 SAVE “EDI” values to Table “EDI” (8) RETURN persistent KSQL query

## 6. Result and Discussion

### 6.1. Output Data and Visualisation

**Figure 17:**
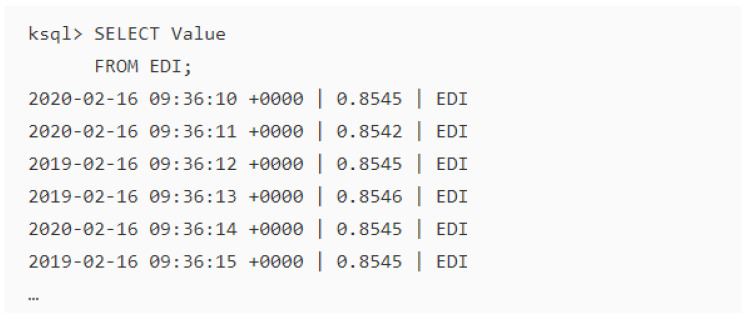
The output stream from the EDI topic in the KSQL editor.

The results of the numerical models executed persistently in the KSQL are committed to the output topic—EDI (Figure 17). The output streams are available through the designated topic and saved the event hub for storage to ensure further availability or visualisation of the data through appropriate plugins. The storage and big data visualisation of the output data streams are outside the scope of this paper. The output value at the time of execution indicates an average EDI value of 0.8545, which signify a near normal drought based on the input values. Further outputs are interpreted by an expert for use by the policy makers for their decision making process. However, to ensure availability of output data streams, distributed database systems provide better performance and offers compatibility across platforms are recommended. Apache Cassandra is a typical example of a highly scalable open-source distributed NoSQL database system that can be used to save the output data streams.

### 6.2. Scalability Of Kafka Cluster

**Figure 18:**
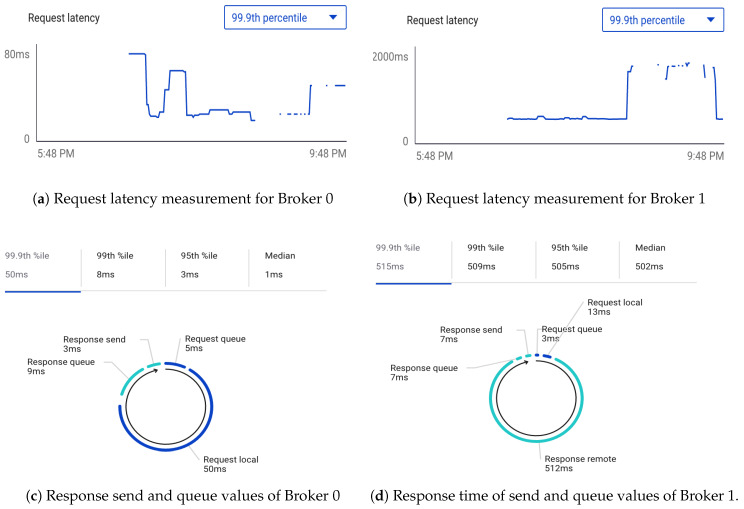
Performance evaluation simulation results of brokers in the cluster.

The result of the performance evaluation of the distributed middleware infrastructure is presented in this section; this determines the real-time effectiveness of the infrastructure. All applications need monitoring. The 99.9 percentile measurement is a total metric that gives a view into the overall performance of requests to the *Kafka* broker. The performance evaluation compares the time it takes the *Kafka* cluster to execute the EPL-based numerical model in the two nodes in the clusters. A critical metric is the request-latency, which provides the average amount of time a produce request sent to the brokers takes to execute. This measurement is the average amount of time in milliseconds. A rise in the request latency value means that produce requests are getting slower, which could be due to networking issues, latency, or broker configuration depending on the implementation platform. However, latency values in milliseconds are within the acceptable limit. In Figure 18, we measure the brokers request latency values (Figure 18a,b), response send, response queue, request local and the total request queue values (Figure 18c,d) of executing the numerical model algorithm. By observing the figure, we notice that our proposed solution through the adoption of Apache *Kafka* provides a high throughput and decreases the processing time for the input data streams. An average response time for 3 ms (Figure 18c) and request latency below 70 ms (Figure 18a) was received for Broker 0. On the other hand, Broker 1 has a median request latency value of 502 ms (Figure 18b), response queue and send value of 7 ms (Figure 18d), all within acceptable values in milliseconds. Our approach enjoys lower runtime considering the heterogeneity of the input data sources. The reason is that, although our solution consists of many phases, adopting a unified data pipeline across the implementation phases reduces the processing time. This is particularly useful in application areas that requires on the spot processing and analysis of huge heterogeneous dataset.

## 7. Conclusions

In this study, we presented the application of a distributed stream processing framework for the real-time big data analysis of heterogeneous environmental management and monitoring data using Apache *Kafka* in Confluent platform. We demonstrate the suitability and applicability of applying big data techniques for processing and analysis of environmental data from heterogeneous systems in real-time, contrary to widely adopted ETL techniques. In particular, we considered the possibility of integrating dataset from legacy systems, external databases with stream processing engine in a unified manner, due to the crucial nature of historical data in environmental modelling. Hence, we introduce the ESTemd framework, which is able to hide the underlying complexities of heterogeneous systems through the use of a unified data pipeline for seamless integration and execution. The application of the framework will improve real-time analytics of environmental data. Furthermore, we have demonstrated the ease of use of this framework to implement a numerical model in the environmental management and monitoring domain. Also, the implementation of the presented ESTemnd framework in a development environment helped to understand how similar implementation should be structured.

Among the future research directions, we would like to investigate the integration of semantic representation layer within the system for more accurate and improved real-time data analytics, also the visualisation of the output data streams using Apache Kibana [61] or Apache Zeppelin [65] with implementation using a complex computational model. We believe that with the open-source availability of the underlying technologies and the extensibility of the ESTemd framework, researchers will be able to extend this framework for the development of real-time stream processing applications in other fields.

## Figures and Tables

**Table 1:** Features of existing stream processing engines, platforms and message brokers.

Features	Storm	Kafka	Samza	Flink	Spark	RabbitMQ
Scalability	Yes	Yes		Yes	Yes	Yes
High availability	High	High	High	High	High	High
Performance	High	Very high	High	High	High	High
Replication	No	Yes	Yes			No
Latency	Low	Low		Low	High	
Cluster Manager	Zookeeper	Zookeeper	YARN	YARN, Mesos	YARN, Mesos	
SQL Querying	No	KSQL	SamzaSQL	No	SparkSQL	No
EP Engine	Yes	Yes		Yes	Yes	
Message Broker	Yes	Yes	No	No	No	Yes
Throughput	High	Very high	Very high	High	High	High

**Table 2:** EDI classification table [66,67].

Drought Classes	Criterion
Extreme Drought	EDI ≤ 2.0
Severe drought	−2.0 ≤ EDI ≤ −1.5
Moderate drought	−1.5 ≤ EDI ≤ −1.0
Near normal drought	−1.0 ≤ EDI ≤ 1.0

**Table 3:** Categorisation of the Sensors Readings to Kafka Topics.

Type of Readings	Kafka Topic
Temperature	TemperatureSensors
Humidity	HumiditySensors
Precipitation	PrecipitationSensors
Atmospheric Pressure	AtmosPressureSensors
Soil Moisture	SoilMoistureSensors
